# *Gordonia sputi* Bacteremia

**DOI:** 10.3201/eid1509.080903

**Published:** 2009-09

**Authors:** Aurélie Renvoise, Jean-Robert Harle, Didier Raoult, Véronique Roux

**Affiliations:** Hôpital de la Timone, Marseille, France (A. Renvoise, D. Raoult, V. Roux); Hôpital de La Conception, Marseille (J.-R. Harle)

**Keywords:** Gordonia sputi, Gordonia spp., bacteremia, immunocompromised host, PCR, bacteria, letter

**To the Editor:** In November 2007, a 69-year-old man with fever was hospitalized at the Northern Hospital in Marseilles, France. He also had diabetes, high blood pressure, and alcohol and tobacco addictions. In September 2007, he had received a diagnosis of laryngeal cancer, which required 2 chemotherapy treatments through a central venous catheter (CVC), the second of which he had received 6 days before his November visit. Prostatic cancer, diagnosed 1.5 years earlier, had been treated by radiotherapy. At the time of the November admission, he had leukopenia (1.77 × 10^9^ leukocytes/L with 0.49 × 10^9^ polymorphonuclear cells/L) and an elevated C-reactive protein level (151 mg/L). The patient was admitted with a preliminary diagnosis of drug-induced febrile granulocytosis; the origin of his fever remained unclear. Blood for culture was first collected from a peripheral vein and on the next day was collected from a peripheral vein and from the CVC. Gram-positive rods grew in the aerobic bottle from the CVC sample. The microorganism was identified by biochemical tests using API Coryne strip (bioMérieux, Marcy-l’Etoile, France) as *Rhodococcus* spp*.* (94% similarity). The day after hospital admission, the patient was empirically treated with intravenous ticarcillin-clavulanate, 5 g 3×/day; ciprofloxacin, 200 mg 2×/day; and granulocyte colony–stimulating factor. One day later, fever resolved and the polymorphonuclear cell count was within normal limits. Oral antimicrobial drug therapy was continued for 1 week.

Bacterial identification of the strain was performed by 16S rRNA sequencing. We obtained a 1,464-bp sequence, which was found to differ at only 2 nt positions from that of *Gordonia sputi* (GenBank accession no. X80634). We concluded that our patient had catheter-related bacteremia caused by *G. sputi* because he was immunocompromised and had a CVC. We ruled out a contaminant because the organism did not belong to the normal flora of human skin and because fever resolved after treatment with antimicrobial drugs.

The genus *Gordona* was first described in 1971, for coryneform bacteria isolated from sputum of patients with pulmonary disease or from soil (*1*). It is a member of the mycolic acid–containing group consisting of genera *Corynebacterium*, *Dietzia*, *Gordonia*, *Mycobacterium*, *Nocardia*, *Rhodococcus*, and *Tsukamurella*. The genus has been revised several times by rearrangements with the genera *Rhodococcus* and *Nocardia*, and the name *Gordona w*as changed to *Gordonia* in 1997. The genus belongs to suborder *Corynebacterineae* within the order *Actinomycetales* and currently contains 27 recognized species; only 7 have been described in human disease. Species are identified by molecular analysis.

*Gordonia* spp. cause a wide spectrum of disease in humans ( *2–10;* Table). Neurologic and vascular infections in immunocompromised and immunocompetent patients have been reported. Cutaneous and respiratory infections, otitis externa, osteitis, and arthritis have reportedly occurred only in immunocompetent patients. Bacteria have most often been isolated from blood samples. Bacteremia has started from underlying disease such as a sequestrated lung (*4*) or acute cholecystis (*5*) or has been related to coronary artery surgery (*2*) and frequently to CVCs (*2**,**3**,**6*)*.* Catheter removal has been recommended for treatment of *Gordonia* spp. infections in children (*6*), but the recommendation varied for adults*.* Six cases of infection in children have been described (*6*), appearing as bacteremia, ventriculitis, and brain abscess.

**Table:** Summary of clinical reports involving *Gordonia* spp.*

*Gordonia* sp.	Clinical findings, by system					
	Vascular	Cutaneous	ENT	Nervous	Osteoarticular	Respiratory
*G. rubripertincta*	Bacteremia (CVC) (*2*)					Lung infection (*2*)
*G. terrae*	Bacteremia (cholecystitis) (*5*); bacteremia (CVC) (*2*); bacteremia (CVC) (*6*)†	Granulomatous skin lesion (*2*); palpebral abscess (*9*); granulomatous mastitis (*7*); mycetoma of the hand (*9*)		Meningitis, brain abscess (*2*); brain abscess (*2*)†		
*G. bronchialis*	Bacteremia (sequestrated lung) (*4*)	Recurrent breast abscess (*7*)		Ventriculitis (intraventricular shunt) (*6*)	Sternal wound infections (*2*)	
*G. polyisoprenivorans*	Endocarditis (CVC) (*3*);† bacteremia (CVC) (*3*)†					
*G. otitidis*	Bacteremia (CVC) (*6*)		Otitis externa (*6*)			Bronchitis (*8*)
*G. sputi*	Bacteremia (CVC) (*2*)†; endocarditis (CVC) (*2*); mediastinitis (surgery) (*2*)					
*G. araii*					Arthritis (bioabsorbable tapered screw) (*10*)	

*ENT, ear, nose, throat; CVC, central venous catheter. Patients were immunocompetent unless otherwise noted. When infection was associated with a medical device, the device is listed in parentheses. †Immunocompromised patient.

Microbiologic diagnosis of *Gordonia* spp. remains difficult. Biochemical profiles can lead to incorrect identification of isolates as *Rhodococcus* spp. (*2*,*4*–*6*,*9*) and sometimes *Corynebacterium* spp. (*3*) or *Nocardia* spp. (*6*). Identification at the genus and species levels is presently obtained by 16S rRNA sequence comparisons.

No recommendation for antimicrobial drug susceptibility testing has been unanimously approved, but these microorganisms seem to be susceptible to many antimicrobial drugs (*6*). Previous studies suggest a combination of penicillins and aminoglycosides as a suitable therapy for *Gordonia-*related bacteremia (*3*)*.* Carbapenem or fluoroquinolone in combination with an aminoglycoside can also be used (*6*). Antimicrobial drug susceptibility is similar to that of *Rhodococcus* spp., for which *Gordonia* spp. are usually incorrectly identified. However, although vancomycin is often used to treat *Rhodococcus* spp. infections, in a previous study 11% of *Gordonia* spp. isolates were resistant (*6*). Treatment must therefore be evaluated specifically for each patient.

*Gordonia* spp. are environmental bacteria whose implication in human disease seems to be increasing. Phenotypic identification of bacteria included in this genus is difficult, and they are often poorly identified as *Rhodococcus* spp. or *Corynebacterium* spp. Molecular identification of *Gordonia* spp. by using 16S rRNA gene sequence comparison enables their characterization in human disease because the method is more accurate. The fact that these bacteria are often associated with medical devices highlights their role as nosocomial agents. Gram-positive bacilli must, therefore, not be systematically considered as contaminants, especially if associated with medical devices, and should be thoroughly identified by molecular methods in addition to biochemical tests.
